# β-Cryptoxanthin Maintains Mitochondrial Function by Promoting NRF2 Nuclear Translocation to Inhibit Oxidative Stress-Induced Senescence in HK-2 Cells

**DOI:** 10.3390/ijms24043851

**Published:** 2023-02-14

**Authors:** Ye Zhang, Hu Mao, Yanze Li, Yufeng Xiong, Xiuheng Liu, Lei Wang, Zhiyuan Chen

**Affiliations:** Department of Urology, Renmin Hospital of Wuhan University, Wuhan 430060, China

**Keywords:** oxidative stress, senescence, β-Cryptoxanthin, NRF2, HK-2 cells

## Abstract

The mechanisms of acute kidney injury and chronic kidney disease remain incompletely revealed, and drug development is a pressing clinical challenge. Oxidative stress-induced cellular senescence and mitochondrial damage are important biological events in a variety of kidney diseases. As a type of carotenoid, β-Cryptoxanthin (BCX) has various biological functions, which means it is a potential therapeutic candidate for the treatment of kidney disease. However, the role of BCX in the kidney is unclear, and the effect of BCX on oxidative stress and cellular senescence in renal cells is also unknown. Therefore, we conducted a series of studies on human renal tubular epithelial (HK-2) cells in vitro. In the present study, we investigated the effect of BCX pretreatment on H_2_O_2_-induced oxidative stress and cellular senescence and explored the potential mechanism of BCX action. The results showed that BCX attenuated H_2_O_2_-induced oxidative stress and cellular senescence in HK-2 cells. Moreover, BCX promoted NRF2 nuclear expression, maintained mitochondrial function, and reduced mitochondrial damage in HK-2 cells. In addition, silencing NRF2 altered the protective effect of BCX on mitochondria and significantly reversed the anti-oxidative stress and anti-senescence effects of BCX in HK-2 cells. We concluded that BCX maintained mitochondrial function by promoting NRF2 nuclear translocation to inhibit oxidative stress-induced senescence in HK-2 cells. In light of these findings, the application of BCX might be a promising strategy for the prevention and treatment of kidney diseases.

## 1. Introduction

Oxidative stress is the process of oxidative damage caused by the accumulation of free radicals in the body due to the production of large amounts of reactive oxygen species (ROS) [1]. Under physiological conditions, the ROS products are efficiently eliminated by superoxide dismutase (SOD), the glutathione system, catalase, and coenzyme Q, maintaining the balance of redox in the cell [2]. However, when ROS production overcomes the metabolic capacity of the antioxidant defense system, the organ or cell will be in a state of oxidative stress. Additionally, these ROS products can cause oxidative damage, including DNA damage, lipid and protein oxidation, and mitochondrial dysfunction [3]. Oxidative stress is closely associated with the development of many diseases, such as diabetes, atherosclerosis, retinal deformation, and cerebral spongiform malformations [4,5,6,7,8]. In particular, oxidative stress-induced cell damage is involved in the occurrence and development of many kidney diseases, such as acute kidney injury (AKI), diabetic kidney disease, hypertensive nephropathy, renal fibrosis, etc. [9,10,11]. AKI is a worldwide health problem, with a 50% mortality rate for patients with severe AKI [12]. In addition, the global economic burden of chronic kidney disease from all causes continues to increase [13]. In view of these problems, one study found that nuclear factor erythroid 2-related factor 2 (NRF2) activators protect kidney function by reducing oxidative stress, suggesting that reducing oxidative stress is a key factor in the treatment of kidney diseases [14].

Cellular senescence is considered to be a type of stress response, which is typically characterized by stable cell cycle arrest. It can be induced by a variety of intrinsic and extrinsic damages, including oncogenic activation, oxidative and genotoxic stress, mitochondrial dysfunction, radiation, and chemotherapeutic agents [15]. Oxidative stress, in particular, contributes much to cellular senescence in many ways. The cell cycle arrest in senescent cells occurs through two main signaling pathways: RB-p16 and TP53-p21 [16]. The sustained DNA damage response in senescent cells caused an increase in γ-H2AX [17]. In addition, Ki67 is a marker of circulating cells, while pRPS6 is a marker of active protein synthesis. These two markers, together with the assessment of SA-β-galactosidase activity, allow further identification of senescent cells [18]. Previous studies have indicated that cellular senescence promoted a host of degenerative pathologies and that the induction of senescence in cancer cells was an effective means of preventing tumorigenesis [19]. In a recent study [20], Bae et al. found that reducing mitophagy and senescence prevented renal dysfunction caused by AKI. Moreover, other researchers confirmed that inhibition of tubular DNA damage and senescence could attenuate renal interstitial fibrosis after AKI, which was the key point of AKI progression to chronic kidney disease (CKD) [21]. In addition, senescent cells produce a complex secretome, known as the senescence-associated secretory phenotype (SASP), which promotes inflammation and the development of CKD [22]. Therefore, the removal of senescent cells by activating apoptosis appears to be a key mechanism for treating AKI and preventing the progression of CKD. In fact, many efforts have been made to develop new drugs to eliminate senescent cells. However, current research is still very limited.

β-Cryptoxanthin (BCX) is one of the common carotenoids that is widely found in fruits and vegetables such as oranges, red peppers, and pumpkins. As an antioxidant, it has a variety of functions that are important for human health. Early studies showed that BCX had a stimulatory effect on bone calcification and played a vital role in bone homeostasis [23]. There is also evidence that BCX has potentially beneficial effects on the prevention of various cancers through its antioxidant capacity [24]. In addition, a recent study found that BCX alleviated myocardial ischaemia/reperfusion injury by inhibiting NF-κB-mediated inflammatory signaling [25]. While foods with high BCX content may be beneficial in the prevention of kidney diseases, whether BCX can work by attenuating oxidative stress and cellular senescence is still unknown. Therefore, we investigated the effects of BCX pretreatment on H_2_O_2_ -induced oxidative stress and senescence in human renal tubular epithelial cells. In addition, the nuclear translocation of NRF2 regulates mitochondrial redox homeostasis and is an important protective mechanism to maintain mitochondrial function and resist oxidative stress [26,27]. Therefore, we also investigated the effect of BCX on NRF2 to further explore its specific mechanism of action in renal diseases.

## 2. Results

### 2.1. H_2_O_2_-Induced Oxidative Stress Resulted in Senescence in HK-2 Cells

**Figure 1:**
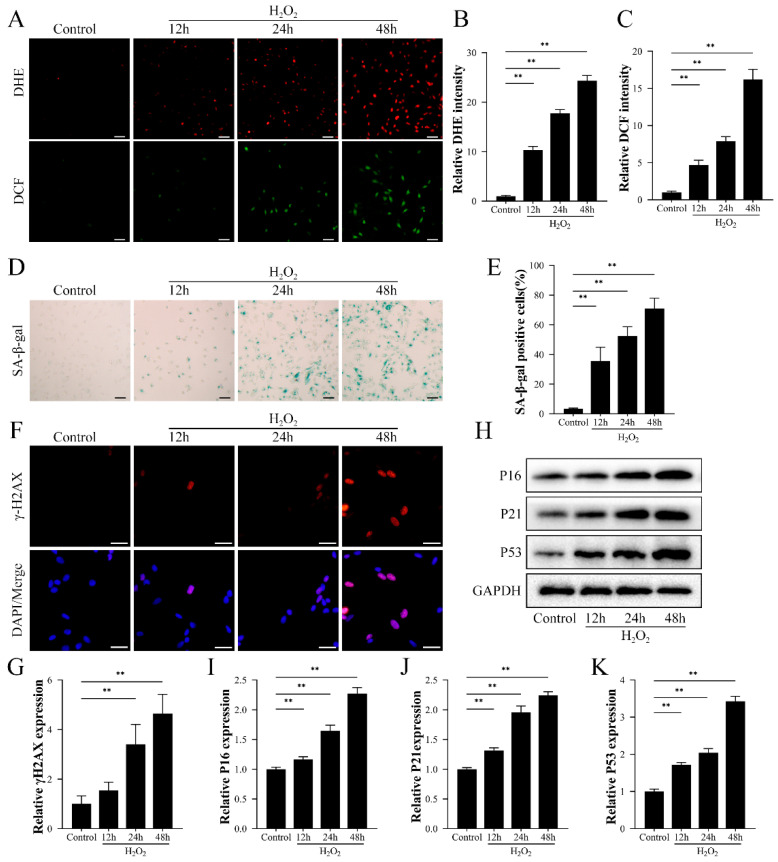
H_2_O_2_-induced oxidative damage induced the expression of senescence-associated factors in HK-2 cells. HK-2 cells were treated with 800 µM H_2_O_2_ for 12, 24, and 48 h. (**A**–**C**) The levels of superoxide anion and reactive oxygen species in HK-2 cells were detected by DHE and DCF staining. Scale bar = 50 μm. (**D**,**E**) Detection of senescence levels in HK-2 cells by SA-β-galactosidase staining. Scale bar = 50 μm. (**F**) Immunofluorescence detection of cellular senescence marker γ-H2AX expression. (**G**–**K**) Western blot detection of expression of senescence-related proteins P16, P21, and P53. Values are expressed as mean ± SD. The mean value is the average of the results of three replicate experiments. *n* = 3. ** *p* < 0.01.

To investigate the effect of H_2_O_2_-induced oxidative stress on cellular senescence, HK-2 cells were treated with H_2_O_2_ for 12, 24, and 48 h. DHE and DCF staining results showed that the levels of superoxide anion and ROS increased with increasing time of H_2_O_2_ treatment, indicating that H_2_O_2_ treatment could induce oxidative stress in HK-2 cells (Figure 1A–C). In addition, SA-β-gal staining results suggested that H_2_O_2_ could induce senescence in HK-2 cells (Figure 1D,E). As shown in Figure 1H–K and Supplementary Figure S1, H_2_O_2_ increased expression of senescence-related proteins such as P16, P21, P53, RB, RB2, P107, P27 and ARF and also caused an increase in the level of γ-H2AX (a cellular senescence marker) in a time-dependent manner (Figure 1F,G). The above results indicated that the oxidative stress caused by H_2_O_2_ treatment could induce HK-2 cell senescence and identified H_2_O_2_ incubation for 48 h as the condition for constructing the senescence model in subsequent experiments.

### 2.2. BCX Attenuated H_2_O_2_-Induced Senescence in HK-2 Cells

**Figure 2:**
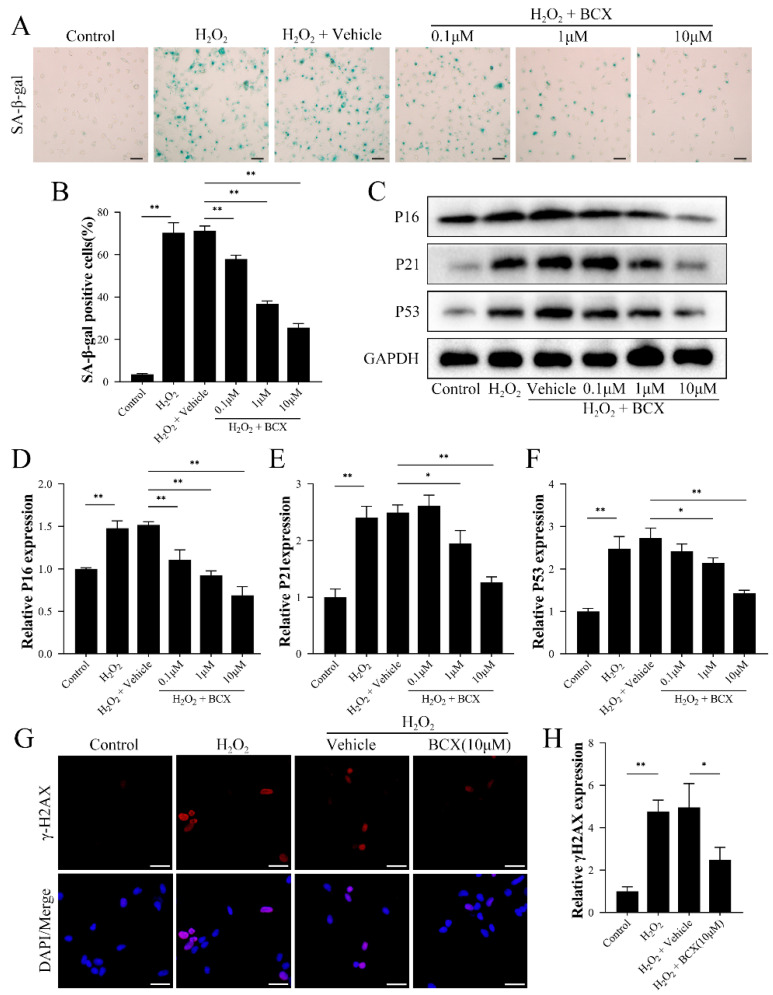
BCX inhibited oxidative stress-induced senescence in HK-2 cells in a concentration-dependent manner. After pretreatment with different doses of BCX (0.1, 1 and 10 µM) for 24 h, HK-2 cells were treated with 800 µM H_2_O_2_ for 48 h. (**A**,**B**) Senescence levels of HK-2 cells were detected by SA-β-galactosidase staining. Scale bar = 50 μm. (**C**–**F**) Western blot detection of expression of senescence-associated proteins P16, P21, and P53. The quantitative analysis was expressed as a relative value to GAPDH. (**G**,**H**) Immunofluorescence detection of the expression of cellular senescence marker γ-H2AX. Scale bar = 40 μm. Values are expressed as mean ± SD. The mean value is the average of the results of three replicate experiments. *n* = 3. * *p* < 0.05 and ** *p* < 0.01.

To explore the effect of BCX on the senescence of HK-2 cells, we added different concentrations of BCX to the HK-2 cells before the senescence model was constructed by H_2_O_2_. As shown in Figure 2A,B, SA-β-gal-positive cells were significantly increased after H_2_O_2_ treatment, while BCX was able to significantly reduce SA-β-gal-positive cells in a dose-dependent manner. In Supplementary Figure S3, we examined the expression of Ki67 and pRPS6. These results further clarified that BCX ameliorates the transition of circulating cells to senescent cells caused by H_2_O_2_. In addition, BCX addition inhibited the increase in expression of proteins P16, P21, and P53 caused by H_2_O_2_ treatment in a dose-dependent manner (Figure 2C–F). Similarly, BCX pretreatment reduced the expression of several other senescence-associated proteins (Supplementary Figure S2). Furthermore, as Figure 2G,H shows, the level of γ-H2AX was increased in H_2_O_2_-treated cells compared with the control; BCX addition partially ameliorated the senescence of HK-2 cells caused by H_2_O_2_ treatment.

### 2.3. BCX Inhibited Oxidative Stress in HK-2 Cells

**Figure 3:**
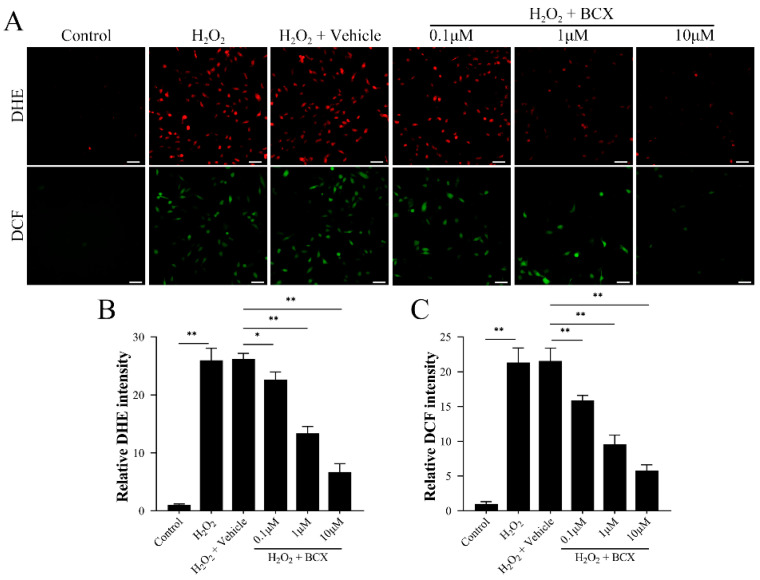
BCX significantly suppressed H_2_O_2_-induced oxidative stress in HK-2 cells. After pretreatment with different doses of BCX (0.1, 1 and 10 µM) for 24 h, HK-2 cells were treated with 800 µM H_2_O_2_ for 48 h. (**A**–**C**) Levels of superoxide anion and reactive oxygen species in HK-2 cells were detected by DHE and DCF staining. Scale bar = 50 μm. Values are expressed as mean ± SD. The mean value is the average of the results of three replicate experiments. *n* = 3. * *p* < 0.05 and ** *p* < 0.01.

To determine whether BCX regulated HK-2 cell senescence by inhibiting the H_2_O_2_-induced oxidative stress pathway, we further examined the indicators related to oxidative stress and found that the levels of superoxide anion and ROS induced by H_2_O_2_ decreased with the increase in BCX dose (Figure 3A–C). This demonstrated that BCX attenuates the oxidative stress induced by H_2_O_2_ in HK-2 cells in a dose-dependent manner.

### 2.4. BCX Promoted NRF2 Nuclear Expression, Maintained Mitochondrial Function, and Reduced Mitochondrial Damage

**Figure 4:**
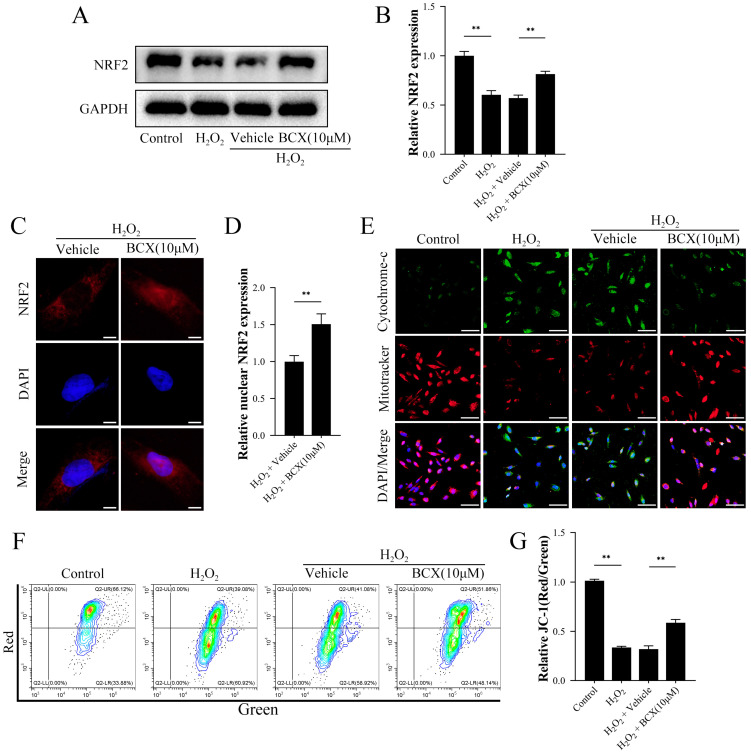
BCX promoted nuclear translocation of NRF2 and maintained mitochondrial function. After pretreatment with BCX (10 µM) for 24 h, HK-2 cells were treated with 800 µM H_2_O_2_ for 48 h. (**A**,**B**) Western blot detection of NRF2 protein expression in HK-2 cells. The quantitative analysis was expressed as a relative value to GAPDH. (**C**,**D**) Immunofluorescence detection of nuclear translocation of NRF2 in HK-2 cells. Scale bar = 5 μm. (**E**) Immunofluorescence of cytochrome C and Mitotracker staining to label mitochondria. Scale bar = 80 μm. (**F**,**G**) Flow cytometry detection of mitochondrial membrane potential levels in HK-2 cells. Values are expressed as mean ± SD. The mean value is the average of the results of three replicate experiments. *n* = 3. ** *p* < 0.01.

Previous research demonstrated that BCX attenuates cardiac ischemia-reperfusion injury by reducing mitochondrial dysfunction in mice [28]. NRF2 is an essential antioxidant molecule that acts as a key regulator between the cellular antioxidant response and energy metabolism [29]. To identify whether BCX can reverse oxidative stress-induced senescence in HK-2 cells by affecting mitochondrial function through NRF2, we first evaluated the effect of BCX on the expression of NRF2 in HK-2 cells. NRF2 expression was reduced in HK-2 cells after H_2_O_2_ treatment compared to the control group. However, BCX treatment significantly increased the expression of NRF2, indicating that NRF2 had a significant role in this process (Figure 4A,B). Subsequent immunofluorescence results further revealed that BCX could also promote the translocation of NRF2 to the nucleus (Figure 4C,D). In addition, BCX ameliorated the mitochondrial damage caused by H_2_O_2_ treatment, as evidenced by an increase in mitochondrial membrane potential, a decrease in the release of cytochrome-c, and a restoration of mitochondrial activity (Figure 4E–G). These results together suggested that BCX exerts a mitochondrial protective effect by promoting the translocation of NRF2 to the nucleus.

### 2.5. Silencing NRF2 Reverses the Protective Effect of BCX on Mitochondria and Promotes Oxidative Stress

**Figure 5:**
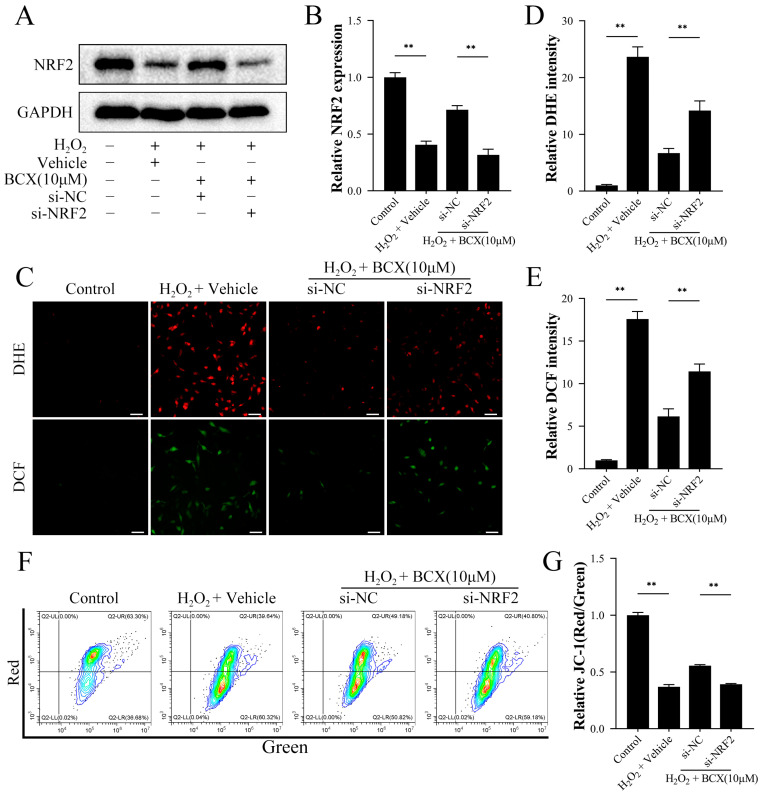
Inhibition of NRF2 reversed the protective effect of BCX against oxidative stress and restored mitochondrial membrane potential. HK-2 cells were transfected with negative control or si-RNA for NRF2 (si-NRF2). After transfection, HK-2 cells were pretreated with or without BCX (10 µM) for 24 h and treated with 800 μM H_2_O_2_ for 48 h. (**A**,**B**) Western blot detection of NRF2 protein expression in HK-2 cells. The quantitative analysis was expressed as a relative value to GAPDH. (**C**–**E**) The levels of superoxide anion and reactive oxygen species in HK-2 cells were detected by DHE and DCF. Scale bar = 50 μm (**F**,**G**) Flow cytometry detection of mitochondrial membrane potential levels in HK-2 cells. Values are expressed as mean ± SD. The mean value is the average of the results of three replicate experiments. *n* = 3. ** *p* < 0.01.

To further validate the role of NRF2 in the protection of mitochondrial function and mitigation of oxidative stress by BCX, NRF2 siRNA was used to silence the expression of NRF2 in HK-2 cells. Figure 5A,B shows that the expression of NRF2 was not increased in NRF2-silenced HK-2 cells after the addition of BCX, which demonstrated the knockdown efficiency of NRF2 siRNA. In the meantime, silencing NRF2 reduced the mitochondrial membrane potential, which reversed the protective effect of BCX on mitochondria (Figure 5F,G). Consistent with previous results, BCX significantly reduced the level of H_2_O_2_-induced oxidative stress. Conversely, NRF2 silencing augmented oxidative stress in HK-2 cells, as manifested by an increase in superoxide anions and ROS (Figure 5C–E). These data suggested that BCX maintained mitochondrial function and attenuated oxidative stress through the NRF2 pathway.

### 2.6. Silencing NRF2 Reversed the Anti-Senescence Effect of BCX on HK-2 Cells

**Figure 6:**
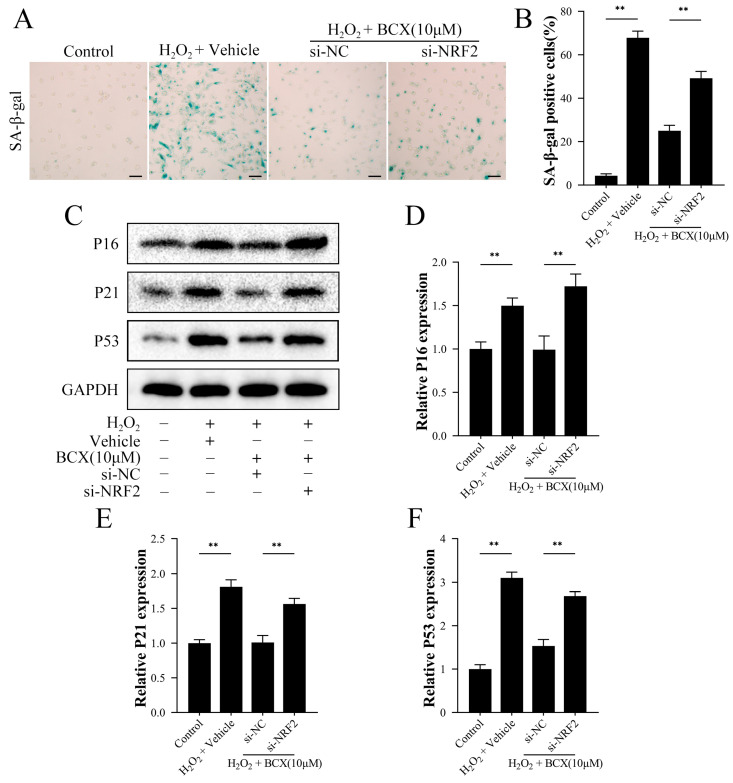
Inhibition of NRF2 reversed the ability of BCX to resist oxidative stress-induced senescence. HK-2 cells were transfected with negative control or si-RNA for NRF2 (si-NRF2). After transfection, HK-2 cells were pretreated with or without BCX (10 µM) for 24 h and treated with 800 μM H_2_O_2_ for 48 h. (**A**,**B**) Senescence levels of HK-2 cells were detected by SA-β-galactosidase staining. Scale bar = 50 μm. (**C**–**F**) Western blot detection of protein expression of senescence-associated proteins P16, P21, and P53. The quantitative analysis was expressed as a relative value to GAPDH. Values are expressed as mean ± SD. The mean value is the average of the results of three replicate experiments. *n* = 3. ** *p* < 0.01.

To establish the role of NRF2 in BCX in reducing the senescence of HK-2 cells, we also treated HK-2 cells with NRF2 siRNA. As shown in Figure 6A,B, BCX significantly reduced the number of SA-β-gal-positive cells in the H_2_O_2_-induced senescence model. However, NRF2 siRNA treatment inhibited the effect of BCX on the clearance of senescent cells. Consistent with previous results, we found that the addition of BCX to H_2_O_2_-induced senescent cells significantly reduced the expression of senescence-associated proteins P16, P21, and P53. However, silencing NRF2 blocked the downregulation of senescence-associated proteins induced by BCX treatment (Figure 6C–F).

## 3. Discussion

The present study focused on the effect of BCX on oxidative stress-induced senescence in renal cells and the possible mechanisms that mediated this effect. We first verified that H_2_O_2_-induced oxidative stress could cause HK-2 cell senescence. Based on the above results, we established the senescent cell model and explored the role of BCX. We first revealed that BCX attenuated HK-2 cell senescence, which was induced by oxidative stress. Further exploring the mechanism, we also found that BCX was able to promote nuclear translocation of NRF2, which consequently attenuated oxidative stress and maintained mitochondrial function. In addition, NRF2 silencing inhibited the role of BCX in anti-oxidative stress and protection of mitochondria, and ultimately led to HK-2 cell senescence. Therefore, our study demonstrated that BCX inhibited HK-2 cell senescence by promoting nuclear translocation of NRF2 to attenuate oxidative stress.

Many studies have shown that oxidative stress is a major factor in the occurrence and development of many kidney diseases [2,30]. As a result of oxidative stress, the uncontrolled increase in ROS leads to lipid peroxidation and protease activation that not only aggravates acute renal injury but also promotes the progression of chronic kidney disease by inducing inflammation [31,32]. In the present study, HK-2 cells were treated with H_2_O_2_ in vitro to mimic the oxidative stress state of the kidney. Previous studies used H_2_O_2_ as a means to induce senescence in HK-2 cells [33]. Similarly, our results demonstrate that H_2_O_2_ treatment induced cellular senescence along with oxidative stress. Furthermore, the degree of senescence was positively correlated with the duration of H_2_O_2_ treatment. Therefore, 48 h was identified as the optimal time in the follow-up study.

Senescence is closely related to a variety of kidney diseases, and measures targeting senescence can be used as a potential treatment for kidney disease. Bae et al. found that Paricalcitol reduced mitochondrial damage and cellular senescence, and consequently attenuated contrast-induced acute kidney injury [20]. Another study revealed that lipofuscin A4 restored septic kidney function by blocking the crosstalk between inflammation and premature senescence [34]. In addition, inhibition of senescence has been shown to alleviate renal interstitial fibrosis after AKI [21,35]. BCX is a natural antioxidant that functions as an antiinflammation and anticancer agent [36]. A recent study noted that BCX improved muscle atrophy in the senescence-accelerated mouse-prone 1 mice, suggesting that BCX had anti-senescence effects [37]. In this study, we found that BCX reduced the number of SA-β-gal-positive cells, inhibited the expression of P16, P21, and P53, and reduced the production of the cellular senescence marker γ-H2AX in HK-2 cells. As expected, BCX partially reversed the senescence of HK-2 cells caused by H_2_O_2_. Therefore, we first found that BCX was able to alleviate the senescence of renal cells, promising a wider range of applications.

Increased oxidative stress is a crucial factor in the progression of senescence [38]. We demonstrated the ability of BCX to reduce the level of H_2_O_2_-induced oxidative stress in HK-2 cells. As the central regulator of cellular resistance to oxidative stress, NRF2 translocates into the nucleus under oxidative stress and then enhances the function of the antioxidant defense system [26,39]. The increased ROS in mitochondria damages mitochondrial DNA, and causes leakage of cytochrome-c in mitochondria, which can exacerbate the disruption of intracellular energy metabolism and ultimately lead to cellular necrosis and apoptosis [40]. Studies have shown that NRF2 regulates mitochondrial redox homeostasis in several forms to ensure normal cellular function [27]. A previous study found that BCX decreased visceral fat and cardiometabolic health risk factors by regulating NRF2 pathways [41]. Consistent with previous studies, our study found that BCX increased the expression of NRF2 in HK-2 cells under oxidative stress conditions and promoted the translocation of NRF2 to the nucleus. The mitotracker is able to specifically label biologically active mitochondria in cells with a fluorescence intensity that is representative of mitochondrial damage and mitochondrial function. We found that the mitotracker staining in H_2_O_2_- and vehicle-treated cells was decreased significantly compared to control. In addition, BCX increased mitochondrial membrane potential and decreased the expression of cytochrome-c, suggesting that BCX ameliorated mitochondrial damage caused by oxidative stress by regulating NRF2. Further silencing experiments verified that NRF2 was the key factor for BCX to maintain mitochondrial function and alleviate oxidative stress in HK-2 cells.

Two important issues with senescence are mitochondrial dysfunction and oxidative stress [42]. While regulating oxidative stress progression and mitochondrial function, NRF2 plays a central role in delaying cellular senescence and preventing age-related diseases [43]. Chen et al. found that 1,25-Dihydroxyvitamin D could upregulate NRF2, inhibit oxidative stress and DNA damage, and inactivate p53-p21 and p16-Rb signaling pathways to exert an antiaging role [44]. Recent studies showed that dapagliflozin, a sodium-glucose cotransporter-2 inhibitor, could prevent the progression of diabetic nephropathy by inhibiting cellular senescence [33]. Further exploration of the mechanism revealed that dapagliflozin reduced oxidative stress and cellular senescence by promoting ketone body-induced NRF2 activation. A report on the aging kidney confirmed that an NRF2 activator ameliorated oxidative stress and mitochondrial dysfunction, which in turn reduced aging-related progressive renal injury [42]. In this study, we revealed that silencing NRF2 reversed the anti-senescence effect of BCX in HK-2 cells, as evidenced by an increase in the number of SA-β-gal-positive cells, senescence-associated protein expression, and the cellular senescence marker γ-H2AX.

The above data suggested that BCX attenuated the senescence caused by H_2_O_2_-induced oxidative stress in renal tubular epithelial cells by promoting NRF2 nuclear translocation. However, our study did not reveal how BCX promotes NRF2 nuclear translocation. On the one hand, BCX may affect the KEAP1-dependent regulation of NRF2 by interacting with KEAP1 proteins; on the other hand, BCX may affect the KEAP1-independent NRF2 regulatory pathway, such as protein kinase C (PKC), c-Jun N-terminal kinase (JNK), or phosphatidylinositol 3-kinase/protein kinase B (PI3K/AKT) pathway [45]. In addition, BCX may also affect the activity of importins while providing a pathway for NRF2 to enter the nucleus. Further exploration is needed to determine the mechanism at the root of this change in the subcellular localization of NRF2 [46]. In any case, we explored the effects of BCX on oxidative stress and senescence in the kidney in vitro. Thus, foods with high BCX content are expected to be used for the prevention of various kidney diseases. In the future, more natural antioxidants will be discovered and applied to the treatment and prevention of kidney diseases.

## 4. Materials and Methods

### 4.1. Extraction and Preparation of BCX

β-Cryptoxanthin (BCX, C6368, TLC ≥ 97%) was purchased from Sigma-Aldrich (Darmstadt, Germany). One milligram of BCX was dissolved in 180.87 μL of dimethyl sulfoxide (DMSO, 471267, Sigma, Darmstadt, Germany) to form the BCX storage solution at a concentration of 10 mM and stored in a refrigerator at −80 °C. The BCX storage solution was thawed in advance before the treatment of HK-2 cells. The Vehicle groups received 0.1% DMSO.

### 4.2. Cell Culture

Human renal tubular epithelial (HK-2) cells were obtained from American Type Culture Collection (ATCC, Rockville, MD, USA). Cells were maintained in Dulbecco’s modified Eagle’s medium (DMEM) (Invitrogen, Karlsbad, CA, USA) supplemented with 10% fetal bovine serum (Gibco, New York, NY, USA). The medium was placed in a 5% CO_2_ and 95% air incubator at 37 °C. After pretreatment with different doses of BCX (0.1, 1 and 10 μM) for 24 h, HK-2 cells were treated with 800 µM H_2_O_2_ for 12, 24, and 48 h to induce oxidative stress.

### 4.3. Western Blot Analysis

HK-2 cells with different treatments were homogenized in RIPA lysis buffer containing protease inhibitors. The protein concentration of each sample was measured by bicinchoninic acid (BCA) assay. The protein content in the gel was 20–30 μg per well. Then, the proteins of each sample were separated by SDS-PAGE and were electrically transferred onto polyvinylidene difluoride transfer membranes. The membranes were then blocked with 5% fat-free milk at normal temperature for 1 h. The blots were incubated with the following primary antibodies overnight at 4 °C: rabbit anti-p53 antibody (Abcam, Cambridge, UK), rabbit anti-p21 antibody (Abcam, UK), rabbit anti-p16 antibody (Abcam, UK), rabbit anti-NRF2 antibody (PROTEINTECH NORTH AMERICA, Chicago, IL, USA) and rabbit anti-GAPDH antibody (Abcam, UK). After incubation with primary antibodies, the blots were washed and incubated with secondary antibodies. Subsequently, the blots were washed three times and visualized by using enhanced chemiluminescence reagents (Thermo Fisher Scientific, Waltham, MA, USA). Finally, the density of each band was quantified by Image-Pro Plus (version 7.0 for Windows, Media Cybernetics, Rockville, ML, USA).

### 4.4. Measurement of Superoxide Anion and ROS Level

Superoxide anion and ROS levels were measured using the dihydroethidium kit (Beyotime Biotechnology, Shanghai, China) and the Reactive Oxygen Species Detection kit (Beyotime Biotechnology, Shanghai, China), respectively. According to the manufacturer’s instruction, HK-2 cells were incubated with dihydroethidium (DHE, 1 μM) or dichlorodihydrofluorescein diacetate (DCF, 10 μM) at 37 °C for 30 min in the medium with DAPI (1 μg/mL, Beyotime Biotechnology, Shanghai, China) in the dark. Then, the cells were observed and photographed under a fluorescence microscope (IX71, OLYMPUS, Tokyo, Japan) after washing.

### 4.5. SA-β-Galactosidase Staining

To detect senescence-associated SA-β-galactosidase (SA-β-gal), HK-2 cells were stained using the SA-β-galactosidase (SA-β-gal) staining kit (Beyotime Biotechnology, Shanghai, China) according to the manufacturer’s instructions. Briefly, HK-2 cells with different treatments were fixed for 10 min, washed three times with PBS, and stained overnight at 37 °C. The next day, the cells were photographed and analyzed under a fluorescence microscope after washing with PBS three times.

### 4.6. Mitotracker Staining

After treatment, HK-2 cells were labeled with Mitotracker Red CMXRos (Beyotime Biotechnology, Shanghai, China) for mitochondria. Cells were incubated with Mitotracker working solution (100 nM) for 20 min at 37 °C and then fixed with 4% paraformaldehyde for 15 min. Immunofluorescence of cytochrome C was continued after three washes with PBS.

### 4.7. Immunofluorescence Staining

Cells with different treatments were fixed with 4% paraformaldehyde for 15 min and washed with PBS three times. After being closed with 10% goat serum, cells were incubated with primary antibody against NRF2 (PROTEINTECH NORTH AMERICA, Chicago, IL, USA), γ-H2AX Ser139 (Abcam, UK), or Cytochrome c (PROTEINTECH NORTH AMERICA, Chicago, IL, USA) overnight. Then, cells were incubated with secondary antibodies with fluorescent labeling (CoraLite488-conjugated Goat Anti-Rabbit IgG, 1:200; Cy3–conjugated Affinipure Goat Anti-Rabbit IgG, 1:100) for 1 h. Finally, using DAPI (Beyotime Biotechnology, Shanghai, China) cell nuclei were labeled before observation. Then, the number of points per cell nuclear region was quantified by Image-Pro Plus.

### 4.8. Small Interfering RNA (siRNA) Transfection

NRF2 siRNA (si-NRF2; CAAACAGAATGGACCTAAA) and negative control siRNA (si-NC; CGAACAGTCACTAGTCACGAT) synthesized by Sangon Biotech were used for transfection. HK-2 cells were inoculated at the appropriate density in six-well plates and cultured for 24 h. Then, transfection was then carried out using Lipofectamine 3000 according to the manufacturer’s instructions. After 24 h, each sample was treated with H_2_O_2_ with or without the addition of BCX and collected for further analysis.

### 4.9. Mitochondrial Membrane Potential (MMP) Assay

HK-2 cells were collected by centrifugation (600× *g*, 4 min) after cell digestion using trypsin. Cells were incubated with JC-1 (Beyotime Biotechnology, Shanghai, China) for 25 min in a 37 °C incubator. After incubation, cells were centrifuged (600× *g*, 4 min) and washed with JC-1 staining buffer, repeating two times. After centrifugation again (600× *g*, 4 min), the cells were resuspended with 0.5 mL of JC-1 staining buffer, and the mitochondrial membrane potential was detected using flow cytometry (CytoFLEX, Beckman Coulter Biotechnology (Suzhou) Co., Ltd., Suzhou, China). JC-1 monomer (Green) was detected using the FITC channel (Ex = 490 nm; Em = 530 nm), and JC-1 polymer (Red) was detected using the PE channel (Ex = 525 nm; Em = 590 nm).

### 4.10. Statistical Analysis

Images were analyzed using Image-Pro Plus. All experiments were repeated at least three times, with at least three replicates of each dataset. The mean value was the average of the results of three replicate experiments. All of the data were expressed by mean value  ±  standard deviation (SD) and analyzed by GraphPad Prism (version 8.0.2 for Windows, GraphPad Software, San Diego, CA, USA). The differences between groups were analyzed through one-way analysis of variance (ANOVA) and the Student–Newman–Keuls test, followed by the Bonferroni post hoc test. *p*  < 0 .05 was considered to be statistically significant.

## 5. Conclusions

Our study revealed the anti-senescence effect of BCX in renal cells, and the protection effect was closely related to the reduction of oxidative stress. In addition, we further explored the mechanism of action of BCX and found that it could improve mitochondrial function and reduce oxidative stress by promoting the nucleation of NRF2. Overall, our study further corroborates previous studies related to the antioxidant and anti-senescence functions of BCX. More importantly, our study provides support for the idea that foods with high BCX content are beneficial in preventing kidney disease, suggesting that BCX has great potential in the treatment of kidney diseases.

## Data Availability

The datasets in this study are available from the corresponding author.

## Supplementary Materials

The following supporting information can be downloaded at: https://www.mdpi.com/article/10.3390/ijms24043851/s1.
