# Supplementary material for: β-Cryptoxanthin Maintains Mitochondrial Function by Promoting NRF2 Nuclear Translocation to Inhibit Oxidative Stress-Induced Senescence in HK-2 Cells
Source: Int J Mol Sci. 2023 Feb 14;24(4):3851. doi: 10.3390/ijms24043851 (PMC9963668; doi:10.3390/ijms24043851)
Supplement: Supplementary file 1 [file ijms-24-03851-s001.zip › ijms-2131366-supplementary.pdf]

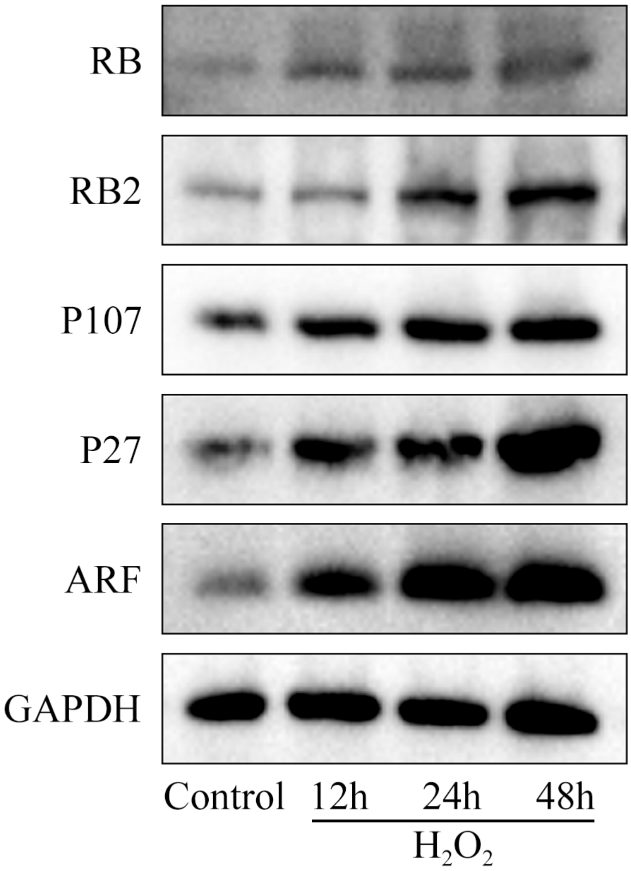

Supplementary Figure S1. The expression levels of senescence-related proteins. HK-2 cells were treated with 800 μM H_2O_2 for 12, 24, and 48 h. Western blot detection of protein expression of senescence-related proteins RB, RB2, P107, P27, and ARF.

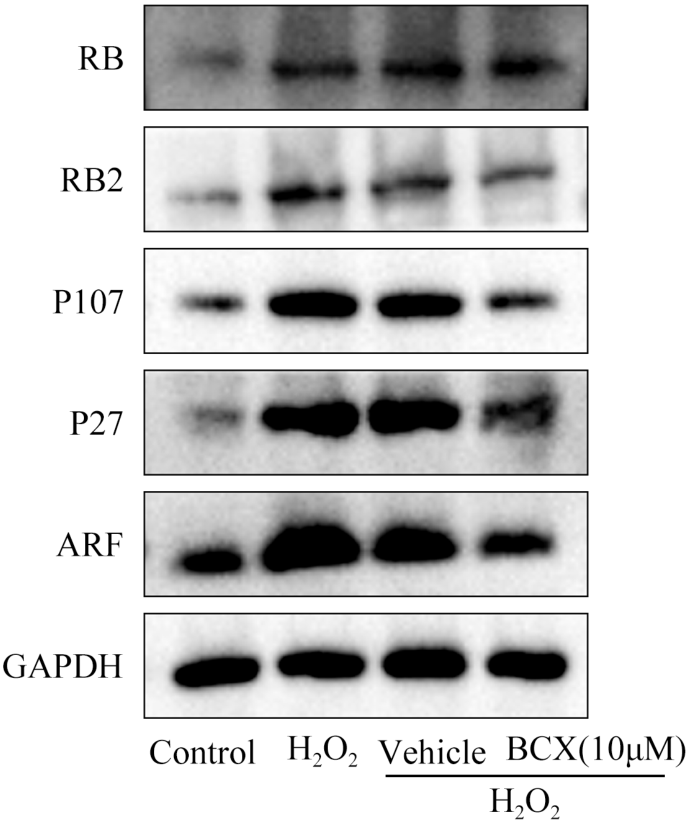

Supplementary Figure S2. The expression levels of senescence-related proteins. After pretreatment with BCX (10 μM) for 24 h, HK-2 cells were treated with 800 μM H_2O_2 for 48 h. Western blot detection of protein expression of senescence-related proteins RB, RB2, P107, P27, and ARF.

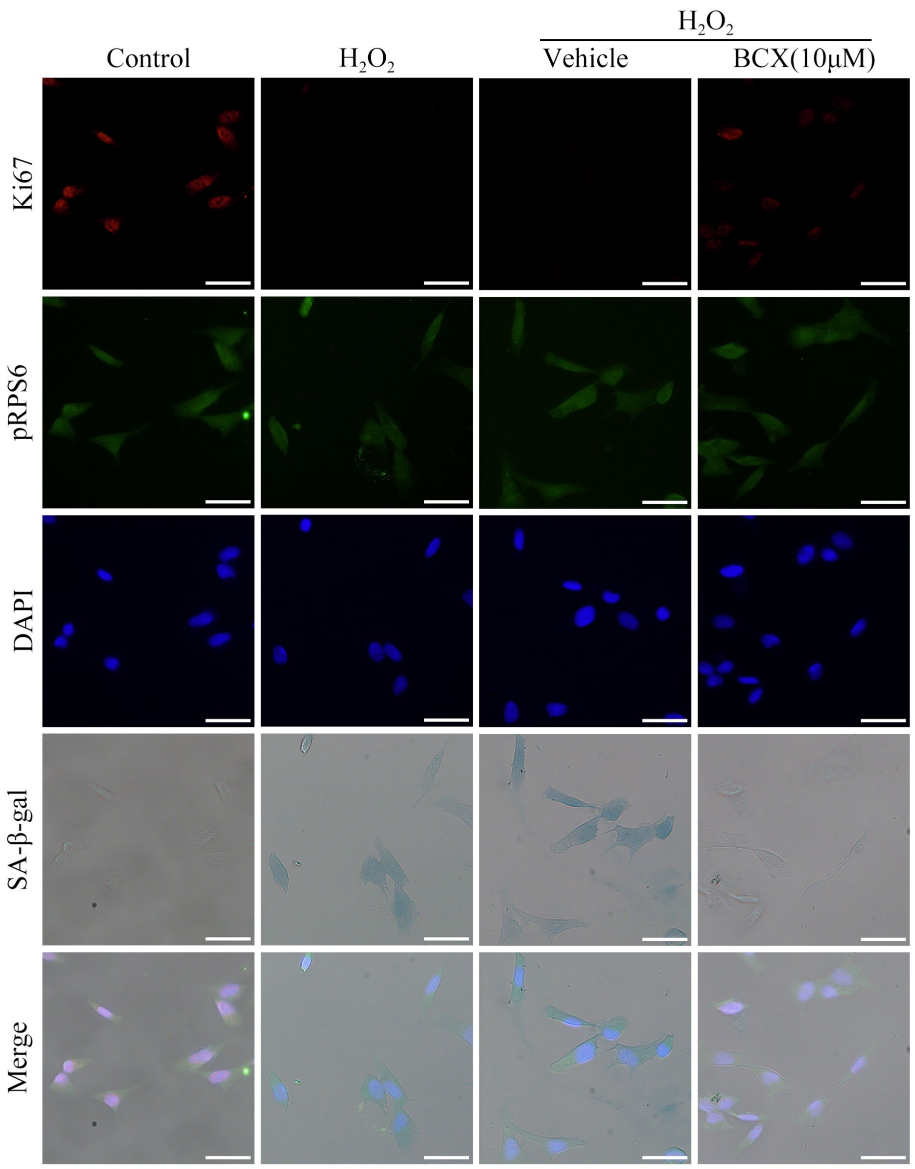

Supplementary Figure S3. The representative images of Ki67, pRPS6, DAPI, and SA-β-galactosidase. After pretreatment with BCX (10 μM) for 24 h, HK-2 cells were treated with 800 μM H_2O_2 for 48 h. SA-β-galactosidase staining was first performed on HK-2 cells. Then the expression of Ki67 and pRPS6 was detected by immunofluorescence technique. Scale bar = 40 μm.
